# Plasma phospho-tau 217 outperforms plasma phospho-tau 181 analyzed with Lumipulse in detecting Alzheimer’s dementia in a real-world memory clinic population

**DOI:** 10.3389/fnagi.2026.1714247

**Published:** 2026-02-13

**Authors:** Michaela Defrancesco, Elke R. Gizewski, Ruth Steiger, Irene Virgolini, Alexander Kroiss, Timo Schurr, Jan Paul Görtz, Josef Marksteiner, Alex Hofer, Christian Humpel

**Affiliations:** 1 Department of Psychiatry, Psychotherapy, Psychosomatics and Medical Psychology, University Hospital of Psychiatry I, Medical University of Innsbruck, Innsbruck, Austria; 2 Department of Radiology, Medical University of Innsbruck, Innsbruck, Austria; 3 Neuroimaging Core Facility, Medical University of Innsbruck, Innsbruck, Austria; 4 Department of Nuclear Medicine, Medical University of Innsbruck, Innsbruck, Austria; 5 Department of Psychiatry and Psychotherapy A, Hall State Hospital, Hall in Tirol, Austria

**Keywords:** Alzheimer’s disease, biomarkers, blood biomarkers, neurocognitive diseases, phospho tau protein

## Abstract

**Introduction:**

We need easy-to-use, valid biomarkers for diagnosing Alzheimer’s disease (AD). Blood biomarkers are a promising option, especially as there is a good correlation with beta-amyloid and tau biomarkers measured by positron emission tomography or in the cerebrospinal fluid. We investigated the added value of measuring these markers in the blood of patients who have already been diagnosed, and how cut-off values can be set in clinical routine.

**Methods:**

Plasma pTau 181 and pTau 217 levels were analyzed using Lumipulse G600II in 132 memory clinic outpatients with different forms of dementia and in 43 cognitively intact controls (CIC). Linear discriminant analysis was used to determine the diagnostic accuracy of pTau 181, pTau 217, and the pTau 217/181 ratio in plasma. The Youden index (YI) was calculated to determine optimal cut-points.

**Results:**

The diagnostic performance of pTau levels to distinguish between dementia patients and CIC yielded an AUC of 0.724 (95% CI: 0.635–0.813) with a cut point (YI) of 1.46 pg/mL for pTau 181 and an AUC of 0.845 (95% CI: 0.763–0.927) with a cut point (YI) of 0.23 pg/mL for pTau 217. The pTau217/181 ratio showed slightly better discriminatory performance (AUC of 0.858 [95% CI: 0.769–0.946]) at a cut point (YI) of 0.18.

**Discussion:**

We suggest that the combined use of pTau217 and pTau181 as a ratio may serve as a valuable method to differentiate AD from non-AD dementia and from cognitively healthy individuals. However, plasma pTau217 outperforms pTau181 and the ratio due to its greater stability and marginally higher sensitivity compared to the 217/181 ratio. Plasma pTau217 levels above the cut-off point could indicate the need for further investigation of biomarkers.

## 1 Introduction

Alzheimer’s disease (AD) is the most prevalent neurodegenerative disease in the older population worldwide. A 55 million people currently suffer from Alzheimer’s disease dementia (ADD). AD begins with a long preclinical phase without clinical symptoms but a measureable increase in neuropathological changes in the brain, such as the deposition of beta-amyloid (Aß) and tau protein. Subsequently, people develop Mild Cognitive Impairment (MCI) and finally ADD with significant cognitive impairment and deficits in daily activities (Sperling et al., 2011). Worldwide, 77 million people are thought to be on the so-called AD continuum, which includes people with positive biomarkers for AD and/or without measurable clinical symptoms (Jack et al., 2024). For a long time, neuropathological changes could only be detected at autopsy. However, there are now biomarkers for accurate diagnosis of AD through the examination of cerebrospinal fluid (CSF) or through Aß or tau positron emission tomography (PET). The first is invasive and the latter expensive and is usually only available in specialized centers. Therefore, PET cannot be used in daily clinical practice for all patients with suspected AD. However, it is becoming apparent that in the near future, blood-based biomarkers will make it possible to diagnose or rule out AD with a high degree of accuracy and ease of use (Palmqvist et al., 2021). Recently, plasma assays of beta-amyloid 42/40 and phospho-tau (pTau) became commercially available and have been evaluated in large study populations (e.g., BioFINDER cohort).^1^ The Fujirebio Lumipulse plasma assays and the Quanterix Simoa assays for pTau 181 and pTau 217 showed a high correlation with Aß and tau pathology measured with PET or in the CSF (Hansson et al., 2023, Ashton et al., 2022, Martínez-Dubarbie et al., 2024, Figdore et al., 2024, Janelidze et al., 2023, Thijssen et al., 2021). Accordingly, these two pTau biomarkers represent a promising approach for the screening and diagnosis of AD in the preclinical and prodromal stages. Numerous studies found a high diagnostic accuracy for AD by calculating the plasma pTau217/Aβ42 ratio (Arranz et al., 2024, Cecchetti et al., 2024, Wang et al., 2025). These cumulative evidence led to the recent FDA (Food and Drug Administration) approval of plasma p-Tau217/Aβ42 ratio on the Lumipulse platform, but not in terms of the pTau217/181 ratio. It also remains unclear whether it is best to use a single or a combination of the two plasma pTau markers (i.e., by calculating the ratio between them).

The growing number of people with dementia and the emerging disease-modifying therapies make the development of such screening tools extremely important. Once approved by the regulatory authorities, these blood-based biomarker tests could be added to common laboratory tests typically used in clinical routine for patients suspected of having dementia. If these biomarkers are positive in patients with a clinical diagnosis of MCI or mild dementia, they could be used to indicate a higher likelihood of AD. They could also be used to select patients for a more detailed evaluation with CSF or Amyloid-PET to provide proof of indication for the new monoclonal antibody treatment. Blood-based biomarkers represent a huge conceptual and practical change in the understanding and diagnostic assessment of AD and it is likely that there will be increasing demand for these new tests (Teunissen et al., 2022). However, their performance as a screening tool in older people with cognitive impairment suggestive of neurodegenerative disease is currently uncertain (Mielke et al., 2023). Although the revised criteria for the diagnosis and staging of AD recently introduced by the National Institute on Aging and the Alzheimer’s Association (Jack et al., 2024) already include plasma pTau217 as an early disease marker, the applicability of plasma tau markers in the real-world population remains unclear. Furthermore, valid cut points for pTau181 and pTau217 measured with different assays for dementia screening in clinical routine are lacking (Lista et al., 2024, Lehmann et al., 2024). Even analysis of large populations and comparing participants who are amyloid PET positive and negative, the cut-off points for pTau217 show high degrees of variation (Liewand Alzheimer’s Disease Neuroimaging Initiative, 2025). Accordingly, the aims of this prospective study in a real-world memory clinic population were (1) to assess the sensitivity and specificity of plasma pTau181 and pTau217 in patients with dementia diagnosed by biomarkers and clinical criteria, (2) to define valid cut points of these pTau biomarkers to differentiate between cognitively intact subjects and patients with clinically and/or biomarker-based diagnosed ADD and non-AD.

## 2 Materials and methods

### 2.1 Study design

This was a prospective study to assess applicability of pTau181, pTau217, and the pTau 217/181 ratio to differentiate between patients with dementia and age – and gender-matched cognitively intact controls (CIC) in a real-world psychiatric memory clinic population. Data were collected from people who had initially visited the memory clinic as part of routine clinical practice between October, 2023 and September, 2024.

Inclusion criteria comprised: age ≥ 55 year, Mini-Mental State Examination (MMSE) ≥ 15, written informed consent and no pre-diagnosed dementia and anti-dementia therapy (cholinesterase inhibitor or memantin). The exclusion criteria comprised decompensated metabolic disease, acute myocardial infarction, acute cancer, severe renal failure, current alcohol abuse or drug dependence, and major neurological or psychiatric diseases such as stroke, epilepsy or schizophrenia.

All participants underwent neuropsychological assessment as well as a clinical interview and provided a blood sample. Information on somatic comorbidities, ApoE ε genotype, and currently prescribed psychotropic and somatic medications was obtained from medical records. Of the 132 patients diagnosed with dementia, 78% underwent magnetic resonance imaging (MRI) and 22% cerebral computed tomography. A subgroup of 62 patients underwent a detailed biomarker evaluation as part of their diagnostic assessment at the memory clinic, with Aβ PET (*n* = 31) or CSF sampling (*n* = 31) depending on availability, the need for a differential diagnosis and patient preference. Data of CIC were obtained from a study population of another local clinical trial but underwent the same neuropsychological and clinical diagnostic assessment at the memory clinic.

Clinical diagnosis of dementia (DEM) was based on ICD-10 criteria and included: (1) the presence of memory impairment and disturbance of other cognitive function domains on the neuropsychological assessment of > 2 standard deviations (SD), corrected for age and education, (2) significant disrupts of everyday life activities, (3) no disturbances in consciousness, (4) one or more emotional symptoms (e.g., emotional lability, irritability, apathy, or disturbed social behavior), (5) cognitive complaints over the previous 6 months reported by the patient and/or an informant, and (6) a Clinical Dementia Rating (CDR) scale score ≥ 1. Frontotemporal dementia was diagnosed if, in addition to ICD-10 criteria, criteria for probable or possible behavioral variant (Rascovsky et al., 2007) or primary progressive aphasia and its variants (Gorno-Tempini et al., 2011) were met. Vascular dementia was diagnosed if, in addition to ICD-10 criteria, criteria for vascular cognitive impairment (Skrobot et al., 2017) were met. Secondary dementia due to psychiatric disorder was based on ICD-10 criteria and if no other form of dementia was likely to be the underlying etiology of the dementia.

ADD criteria (probable or possible dementia due to AD; McKhann et al., 2011) additionally included a positive marker of neuronal injury (atrophy of the temporal lobe corresponding to a medial temporal atrophy (MTA) score ≥ 2. Alzheimer’s dementia with vascular pathology (ADDvas) was diagnosed if cerebrovascular pathology corresponding to a Fazekas score ≥ 2 was present in addition to the AD criteria.

The authors assert that all procedures contributing to this work comply with the ethical standards of the relevant national and institutional committees on human experimentation and with the Helsinki Declaration of 1975, as revised in 2013. All procedures were approved by the Ethics Committee of the Medical University of Innsbruck, Austria (approval number 1046/2018). Written informed consent was obtained from each participant or their legal representative.

### 2.2 Neuropsychological assessment

All participants completed a neuropsychological test battery including subtests out of the “Consortium to Establish a Registry for Alzheimer’s Disease” (CERAD) battery (Rosen et al., 1984). The test battery included tests to assess verbal memory and recognition (word list learning, word list delayed recall, and word list recognition), constructional praxis (figure drawing), figural memory (delayed recall), confrontational object naming (Boston Naming Test [BNT] – short version), verbal fluency (animals/min, s-words/min), and cognitive flexibility (Trail Making Tests A and B), as well as the MMSE (Folstein et al., 1975). Age- and education-corrected z-scores were calculated from these measures.

### 2.3 Questionnaires and scales

The Neuropsychiatric Inventory (NPI) (Cummings, 1997) was used to assess the frequency (range: 0–4 points), the severity (1–3 points), and the emerging caregiver burden (0–5 points) of twelve behavioral and psychological symptoms of dementia (BPSD). Using the CDR, forgetfulness, difficulties in orientation, judgment and problem solving, community affairs, home and hobbies, and care were evaluated by interviewing a caregiver or informant. An algorithm results in CDR scores ranging from 0 to 3 (0, normal cognition; 0.5, mild impairment; 1, mild; 2, moderate; 3, severe dementia). Depressive symptoms were assessed using the 15-items version of the Geriatric Depression Scale (GDS) (Yesavage et al., 1982). GDS questions were answered with “yes” or “no.” The cumulative score is rated on a scoring grid. The grid sets a range of 0–5 as “not depressed,” 6–10 as “mildly depressed,” and 11–15 as “severely depressed.”

### 2.4 Analysis of plasma pTau181 and pTau217 and CSF

The levels of plasma pTau181 and plasma pTau217 were analyzed with the commercial automated Lumipulse G600II (Fujirebio). The Lumipulse assay is an automated roboter platform using an enzymatic light emitting system. This system gives very fast and accurate values within 35 min. The single racks are placed in the system and each unit contains a triple-tube with the antibodies and magnetic beads.^2^ Standard curves and quality controls (QC) were used for the test. The accuracy of the test is > 5%, and all samples were analyzed in singular (Marksteiner et al., 2022).

Five milliliters of venous blood were collected in EDTA tubes (Sarstedt Monovette EDTA K) and the time was noted on a form. Both were sent to the laboratory via a pneumatic post system within 1 h. The sample was processed in the laboratory within 3–4 h of blood collection, and the processing time was noted. The tube was centrifuged for 5 min at 3,000 g at room temperature using a Thermo Scientific Megafuge 8R. Then, the supernatant was carefully pipetted, and 500 μL of plasma was transferred into a Lumipulse Hitachi cup. This was analyzed immediately, or frozen overnight at -20 °C if this was not possible, and analyzed the next day. For stability, plasma (or blood) was processed the same day and stored for 1–3–7 days at room temperature (RT) or -20°C. As a comparative control, CSF levels of pTau217, pTau181, and their ratio as well as Aß-42 and -40, total tau, and pTau181 were analyzed with the Lumipulse system. CSF was collected by lumbal puncture for routine analysis and processed within 2 days at room temperature. The following cut points were used for CSF interpretation: Beta-amyloid-42 pg/mL: ≤ 500 pg/mL, total Tau pg/mL: > 500 pg/mL, pTau181 pg/mL: > 60 pg/mL, ratio (Tau/Aβ42): > 1.0, and ratio (Aβ42/Aβ40): ≤ 0.06 (Grangeon et al., 2016, Humpel, 2011).

### 2.5 Stability analysis of plasma pTau181 and pTau217

For stability analysis of plasma pTau217 and pTau181, blood was centrifuged, plasma aliquot and analyzed immediately or stored at room temperature for 1–3–7 days and then analyzed. For blood stability, 2 blood samples from the same patient were aliquot, one sample was centrifuged and analyzed immediately, and the other 3 blood samples were stored at room temperature for 1, 3, and 7 days and then centrifuged and analyzed.

### 2.6 MRI acquisition

MRI data acquisition (Siemens Skyra/Verio, 3Tesla scanner) used a predefined standardized protocol with a high-resolution T1-weighted 3D MPRAGE, an axial T2-weighted FLAIR sequence, and a DTI sequence. White matter hyperintensities were rated visually using the Fazekas scale (Fazekas et al., 1987) on FLAIR or T2-weighted images. Medial temporal lobe atrophy was visually rated on coronal T1-weighted 3D MPRAGE sequences (Scheltens et al., 1995, Scheltens et al., 1992).

### 2.7 Beta-amyloid PET imaging

Beta-Amyloid imaging was performed with a GE Discovery MI 4 ring PET/CT scanner at 90 minutes after i.v.-injection of 300 MBq ^18^F-florbetaben (Neuraceq^®^, Life Radiopharma GmbH, Berlin). The PET acquisition time was 10 min, a low-dose CT was used for attenuation correction. PET and MR-images were co-registered using the software provided by HERMES Medical Solutions. The nuclear medicine specialists were specially trained to evaluate the PET scans so that they could recognize whether significant amounts of amyloid plaques were present or not. Visual interpretation was made by comparing the activity in cortical gray matter with activity in adjacent cortical white matter. Each of these brain regions, the lateral temporal, the frontal, the posterior cingulate, the precuneus, and the parietal lobes were systematically visually assessed and scored according to the regional cortical tracer uptake. A negative scan indicates a low or no density of cortical Aß plaques, a positive scan indicates moderate to frequent density.

### 2.8 Statistical methods

Statistical analysis was facilitated using *SPSS* (IBM Corp. Released 2023. IBM SPSS Statistics for Windows, Version 29.0.2.0 Armonk, NY: IBM Corp.) and *R* (R Core Team. R: A language and environment for statistical computing. R Foundation for Statistical Computing, Vienna, Austria. 2024, Version 4.4.1)^3^ with *pROC* (Version 1.18.5) (Robin et al., 2011) and Kuhn M. caret (Classification and Regression Training. R package version 6.0–94. 2023).^4^ All random processes (e.g., data splitting, resampling, and bootstrapping) were controlled by setting a fixed seed (set.seed[111]) in *R* to ensure reproducibility. Initially, study participants were grouped (CIC = cognitively intact controls; ADD = Alzheimer’s disease dementia; ADDvas = AD dementia with vascular pathology; DEM = Dementia) based on clinical, neuropsychological, and imaging measures. Demographics within each group were then summarized using descriptive statistics (mean, median, 25th and 75th percentile, SD, count, percentage) and compared by non-parametric Kruskal-Wallis or Mann-Whitney-U test. The normal distribution of the data was verified using the Shapiro–Wilk test.

Next, we evaluated the diagnostic discriminatory power of the phosphorylated tau proteins (pTau181 and pTau217) and their ratio (pTau217/181). First, logistic regression models were created separately for each biomarker to examine their individual predictive power for the outcome variables (e.g., CIC vs. AD/ADDvas or CIC vs. DEM). The predicted probabilities of these models were used to perform Receiver-Operating Characteristic (ROC) analyses. For the ROC analysis, the diagnostic accuracy of the biomarkers was quantified using the area under the curve (AUC) with calculated confidence intervals (95%) using the DeLong method. The Youden index (Sensitivity + Specificity - 1) was used to determine the optimal cut-off for each biomarker. We did a retrospective power analysis for the single ROC curves (see Table 1), resulting in values larger or equal to 95%.

**TABLE 1:** ROC-based thresholds and diagnostic parameters for pTau plasma measures to differentiate cognitively intact controls vs. ADD/ADDvas and DEM patients.

Participants characteristics	pTau plasma measures	pTau (cut point)	AUC [95% CI]	Accuracy^†,^^§^ [95% CI]^†,^^§^	Kappa (κ)^†,^^§^ [95% CI]^†,^^§^	Sensitivity	Specificity	Model. No.
CIC (*n* = 43) vs. ADD/ADDvas (*n* = 94)	pTau181 (pg/mL)	1.48	0.754 [0.661–0.847]	0.747^†^ [0.604–0.859]^†^ 0.772^§^ [0.546–0.927]^§^	0.330^†^ [0.037–0.618]^†^ 0.383^§^ [-0.230–0.810]^§^	87.23	64.29	1
CIC (*n* = 26) vs. ADD/ADDvas (*n* = 74)	pTau217 (pg/mL)	0.23	0.896 [0.823–0.969]	0.826^†^ [0.716–0.930]^†^ 0.845^§^ [0.606–1.000]^§^	0.534^†^ [0.159–0.806]^†^ 0.610^§^ [0.121–1.000]^§^	86.49	84.61	2
	pTau217/181 ratio	0.18	0.907 [0.826–0.989]	0.832^†^ [0.667–0.944]^†^ 0.857^§^ [0.778–0.980]^§^	0.452^†^ [0.000–0.815]^†^ 0.507^§^ [0.000–0.942]^§^	81.08	92.31	3
CIC (*n* = 43) vs. DEM (*n* = 132)	pTau181 (pg/mL)	1.48	0.724 [0.635–0.813]	0.753^†^ [0.655–0.832]^†^ 0.748^§^ [0.675–0.813]^§^	0.054^†^ [-0.068 to 0.256]^†^ 0.027^§^ [-0.135 to 0.285]^§^	80.30	64.29	4
CIC (*n* = 26) vs. DEM (*n* = 99)	pTau217 (pg/mL)	0.23	0.845 [0.763–0.927]	0.787^†^ [0.682–0.872]^†^ 0.801^§^ [0.543–0.983]^§^	0.201^†^ [-0.015 to 0.500]^†^ 0.329^§^ [-0.251 to 0.945]^§^	76.77	84.62	5
	pTau217/181 ratio	0.18	0.858 [0.769–0.946]	0.810^†^ [0.722–0.907]^†^ 0.802^§^ [0.769–0.898]^§^	0.194^†^ [-0.021 to 0.603]^†^ 0.063^§^ [0.000–0.484]^§^	62.63	96.15	6

AUC, area under the curve; ADD, Alzheimer’s disease dementia; ADDvas, Alzheimer’s disease dementia with vascular pathology; DEM, dementia; n = number of study participants^†^ based on bootstrapping (100 repetitions) ^§^ based on k-fold cross-validations (10-fold).

Next, pairwise comparisons of the AUCs were conducted using the DeLong test to assess statistical differences in discriminatory power between the individual models. To correct for multiple comparisons, Benjamini-Hochberg correction was applied.

To check the stability and robustness of the models, k-fold cross-validations (10-fold) and bootstrapping (100 repetitions) were performed. Model performance was assessed using Accuracy, which reflects the overall proportion of correctly classified cases, and Cohen’s Kappa (κ), which accounts for chance agreement and provides a measure of classification consistency, particularly in the presence of imbalanced classes. Confidence intervals (95 %) were determined from the resampling results for both metrics.

The sensitivity analysis was supplemented by summarizing the sensitivity and 1-specificity (false positive rate) for each threshold value in Supplement Tables 1, 2. The results of the ROC analyses were presented graphically, with the curves for pTau181, pTau217 and pTau217/181 combined in one overview.

**TABLE 2:** Clinical and demographic characteristics of the study sample.

Participants characteristics	Comparison				Test statistic^a^		
	CIC *N* = 43		DEM *N* = 132		*Z =*	*U =*	*p*-value
	Mean ± SD or n (%)	Median (Q1-Q3)	Mean ± SD or n (%)	Median (Q1-Q3)			
Age (years)	71.74 ± 8.43	71 (64–80)	77.90 ± 8.39	80 (73–83)	–4.070	1665.0	< 0.001^a^
Education (years)	12.84 ± 3.23	12(11–13)	10.46 ± 2.42	11(8–12)	–4.805	1379.0	< 0.001^a^
Gender (%female)^c^	22(51)	—	86(65)	—	χ^2^ = 2.686		0.073
MMSE total score	28.60 ± 1.05	29(28–29)	20.34 ± 5.78	22(17–25)	–9.586	75.5	< 0.001^a^
Fazekas score^b^	1.37 ± 0.92	1(1–2)	1.50 ± 0.91	1(1–2)	–0.613	837.5	0.540^a^
MTA score^b^	0.75 ± 0.89	1(1–2)	2.22 ± 1.08	2(2–3)	–3.794	403.0	< 0.001^a^
CDR score	0.01 ± 0.08	0(0–0)	1.38 ± 0.66	1(1–2)	–9.939	1.5	< 0.001^a^
GDS-30 total score	6.05 ± 4.99	5(2–8)	10.16 ± 6.24	9(5–15)	–3.550	8.18.5	< 0.001 ^a^
pTau181 pg/mL	1.61 ± 0.73	1.26(1.07–2.17)	2.39 ± 1.27	2.13(1.52–3.02)	-4.495	1541.0	< 0.001^a^
pTau217 pg/mL	0.19 ± 0.16	1.46(0.09–0.22)	0.56 ± 0.39	0.45(0.25–0.69)	-5.556	402.0	< 0.001^a^
pTau217/181 ratio	0.14 ± 0.02	0.09(0.08–0.15)	0.21 ± 0.09	0.21(0.15–0.26)	–5.746	370.0	< 0.001^a^
ApoE ε4 carrier (%yes)^c^	8(19)	—	61(46)	—	χ^2^ = 12.597		< 0.001

Group comparison of cognitively intact controls and clinically diagnosed dementia (DEM) patients. ^a^Due to deviations from a normal distribution the Mann-Whitney-U-Test was used, CIC, Cognitively intact controls; GDS-30, Geriatric Depression Scale; MMSE, Mini-Mental State Examination; DEM, clinically diagnosed dementia; CDR, linical Dementia Rating scale; MTA, medial temporal atrophy; N, number; SD, standard deviation; Q, quartile. ^b^*N* = 131. ^c^Percentages are rounded.

Additionally, we analyzed the association of p-tau biomarkers with age, the MMSE, and the ApoE ε genotype in patients with ADD (ADD/ADDvas group). To this end, the Spearman correlation for metric variables was employed.

Linear Discrimination Analysis (LDA) is a widely used supervised method for classifying data into distinct groups. It operates by creating a linear combination of predictive variables to classify observations into two or more categories (Gaber et al., 2017, Riffenburgh, 2012). In this study, LDA was applied to classify 98 patients into the two groups ADD/ADDvas and CIC based on demographic, clinical, imaging, and p-tau biomarkers. Patients on one side of the hyperplane are classified as likely belonging to one group, while those on the other side are classified into the opposite group.

To evaluate the potential added value of combining the demographic, imaging, and clinical parameters with p-tau biomarkers, we created a composite model. This model included age, gender, MMSE total score, MTA score, and additionally pTau181, pTau217, and their ratio as predictors. The diagnostic performance of this combined model was evaluated based on sensitivity, specificity, and accuracy.

## 3 Results

### 3.1 Demographics

A total of 175 participants (43 cognitively intact controls, 132 with dementia) were included in this study. Among the patients with dementia, 51 had ADD, 43 ADDvas, and 38 dementia of non-AD etiology (10 vascular dementia, 21 frontotemporal dementia, 6 secondary dementia due to psychiatric disorder). Mean age of the study population was 76.39 ± 8.79 years (61.7% female). Of the 132 patients with dementia, 79% were in a mild stage (MMSE ≥ 20) and 21% in a moderate stage (MMSE 15–9). Detailed subject characteristics are presented in Tables 2, 3.

**TABLE 3:** Clinical and demographic characteristics of the study sample.

Participants characteristics	Diagnostic group of participants			Comparison			
	CIC *N* = 43	ADD *N* = 51	ADDvas *N* = 43	Test statistic^a^	*p*-value	df	*Post-hoc* test^b^
Age (years)	71.74 ± 8.43	76.51 ± 9.27	81.56 ± 5.04	*H* = 27.464	< 0.001^a^	2	CI < ADvas***, CI < AD**, AD < ADvas**
Education (years)	12.84 ± 3.23	10.54 ± 2.21	10.33 ± 2.38	*H* = 22.436	< 0.001^a^	2	CI < AD***, CI < ADvas***
Gender (%female)^c^	22(51)	30(59)	36(84)	χ^2^ = 10.955	0.004	2	
MMSE total score	28.60 ± 1.05	18.34 ± 6.70	20.37 ± 5.35	*H* = 84.266	< 0.001^a^	2	CI < AD***, CI < ADvas***
Fazekas score^d^	1.37 ± 0.92	0.78 ± 0.42	2.16 ± 0.82	*H* = 67.610	< 0.001^a^	2	AD < ADvas***
MTA score^d^	0.75 ± 0.89	2.50 ± 0.91	2.63 ± 0.79	*H* = 17.175	< 0.001^a^	2	CI < AD***, CI < ADvas***
CDR score	0.01 ± 0.08	1.57 ± 0.76	1.38 ± 0.67	*H* = 86.929	< 0.001^a^	2	CI < AD***, CI < ADvas***
GDS-30 total score	6.05 ± 4.99	10.00 ± 5.65	7.51 ± 5.37	*H* = 8.767	0.012	2	CI < AD**
pTau181 pg/mL	1.61 ± 0.73	2.51 ± 1.26	2.35 ± 1.01	*H* = 22.337	< 0.001^a^	2	CI < AD***, CI < ADvas***
pTau217 pg/mL	0.19 ± 0.16	0.65 ± 0.42	0.59 ± 0.41	*H* = 35.999	< 0.001^a^	2	CI < AD***, CI < ADvas***
pTau217/181 ratio	0.14 ± 0.02	0.25 ± 0.08	0.24 ± 0.08	*H* = 39.714	< 0.001^a^	2	CI < AD***, CI < ADvas***
ApoE ε4 carrier (% yes)^c^	8(19)	30(59)	18(42)	χ^2^ = 17.187	< 0.001	2	

Group comparison of cognitively intact controls and patients with Alzheimer’s dementia (ADD/ADDvas). ^a^Due to deviations from a normal distribution the Kruskal-Wallis-Test was used, GDS-30, Geriatric Depression Scale; MMSE, Mini-Mental State Examination; CIC, Cognitively intact controls; ADD, Alzheimer’s disease dementia; ADDvas, Alzheimer’s disease dementia with additional vascular pathology; CDR, Clinical Dementia Rating scale; MTA, medial temporal atrophy; N, number; SD, standard deviation. ^b^Dunn-Bonferroni-Test corrected for multiple comparison. ^c^Percentages are rounded. ^d^*N* = 98. **p < 0.01, ***p < 0.00.

### 3.2 Group comparison of clinical, biological, and demographic data

Patients suffering from dementia were significantly older, displayed more mesio-temporal atrophy, a more pronounced cerebrovascular pathology, and were above all cognitively worse compared to cognitively intact subjects. Gender distribution analysis revealed no statistically significant differences across the study groups. Comparative analysis between patients with ADD and those with ADD with cerebrovascular pathology (ADDvas) demonstrated that the ADDvas cohort presented with significantly elevated Fazekas scores (*p* < 0.01), indicative of more extensive white matter hyperintensities, as well as advanced age (mean difference: 4.3 years, *p* < 0.05). Of the 132 patients with dementia, all patients diagnosed with ADD/ADDvas (*n* = 94) and 13 patients with dementia of non-AD etiology had an MTA score ≥ 2. The latter were all diagnosed with frontotemporal dementia. These findings are comprehensively detailed in Tables 2, 3.

### 3.3 Plasma pTau levels in cognitively intact controls versus dementia patients

Plasma pT181 was significantly higher (1.4-fold) in dementia patients (*n* = 132) compared to controls (*n* = 43). Similarly, plasma pTau217 (2.9-fold) and the pTau217/181ratio (1.5-fold) were significantly higher in patients (Table 2). Comparisons of the pTau181, pTau217 and the pTau217/181 ratio showed no significant differences between patients in the mild and moderate stages of dementia.

### 3.4 Plasma pTau levels in cognitively intact controls versus ADD patients

Plasma pTau181 (1.6-fold), plasma pTau217 (3.4-fold), and the pTau217/181 ratio (1.8-fold) were significantly higher in ADD patients (*n* = 51) compared to CIC (*n* = 43) (Table 3). Similarly, ADDvas patients showed significantly higher plasma levels of pTau181, pTau217 and their ratio (Table 3). Additional correlation analyses between pTau biomarkers and age, the MMSE, and ApoE ε genotype in patients with ADD/ADDvas showed a positive correlation of pTau181 with a higher number of ApoE ε4 allels but no other variables (see Supplementary Material 3).

### 3.5 Plasma pTau in patients with ADD and dementia solely based on clinical measures

The diagnostic performance of pTau plasma measures (181 and 217) and their ratio (217/181) was evaluated for their ability to differentiate between CIC, ADD/ADDvas, and clinically diagnosed dementia (DEM). In the comparison of cognitively intact individuals vs. ADD/ADDvas patients, pTau181 demonstrated moderate discriminatory ability, achieving an AUC of 0.754 (95% CI: 0.661–0.847) at a threshold of 1.48 pg/mL, with sensitivity of 87.2%, specificity of 64.3%, and accuracy of 74.7–77.2%. The Kappa value of 0.33–0.38 indicated slight agreement beyond chance. In contrast, pTau217 performed significantly better, with an AUC of 0.896 (95% CI: 0.823–0.969) at a threshold of 0.23 pg/mL, accuracy of 82.6% – 84.5%, and a Kappa of 0.53–0.61 (moderate agreement). DeLong’s test confirmed that the AUC of pTau217 was significantly higher than that of pTau181 (*p* = 0.019, BH-adjusted *p* = 0.029). The pTau217/181 ratio achieved the highest AUC of 0.907 (95% CI: 0.826–0.989) at a threshold of 0.18, with an accuracy of 83.2–85.7% and specificity of 92.3%, slightly higher discriminatory performance. When comparing pTau217 to the pTau217/181 ratio, however, the difference in AUC was not statistically significant (*p* = 0.655), suggesting comparable diagnostic performance between these two biomarkers.

**FIGURE 1:**
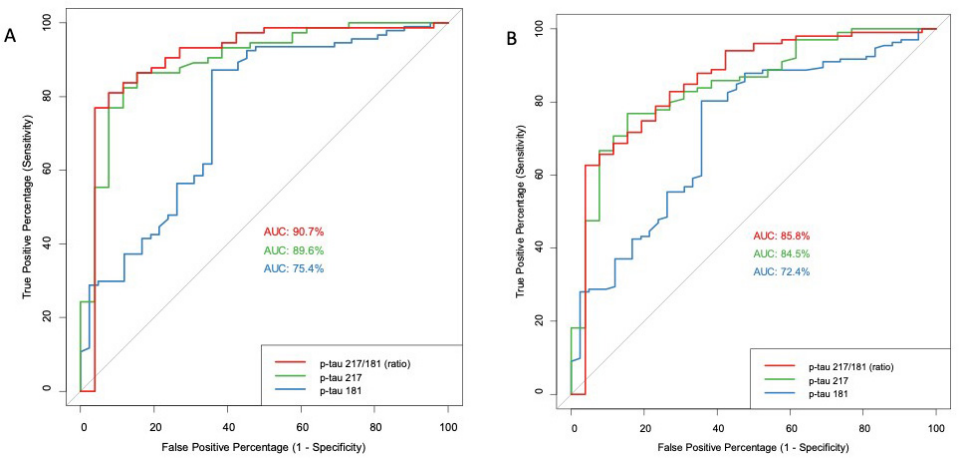
ROC curve for the diagnostic discriminatory power of phospho-Tau (pTau) biomarkers (217, 181, and ratio) for cognitively intact controls versus Alzheimer’s disease dementia (ADD)/Alzheimer’s disease dementia with vascular pathology (ADDvas) patients **(A)** and cognitively intact controls versus dementia (DEM) patients **(B)**.

For the comparison of CIC vs. DEM, pTau181 again showed moderate performance, achieving an AUC of 0.724 (95% CI: 0.635–0.813) at the same threshold of 1.48 pg/mL, with an accuracy of 75.3–74.8% and a Kappa value of 0.03 to 0.05, indicating only slight agreement. pTau217 demonstrated improved performance, with an AUC of 0.845 (95% CI: 0.763–0.927), accuracy of 78.7–80.1%, and a Kappa of 0.20–0.33, reflecting better but still modest agreement. The pTau217/181 ratio again achieved the highest AUC of 0.858 (95% CI: 0.769–0.946), accuracy of 80.1%, and a specificity of 96.2%, underscoring its ability to correctly exclude false positives. DeLong’s test showed a statistically significant improvement in AUC for the pTau217/181 ratio over pTau181 (*p* = 0.038, BH-adjusted *p* = 0.078), but no significant difference was found when comparing pTau217 to the ratio (*p* = 0.681). Detailed results and ROC curves are presented in Table 1 and Figure 1 (for detailed specification of logistic regression models, see Supplementary Material 4).

### 3.6 Stepwise linear discriminant analysis

Linear discriminant analysis (LDA) incorporating demographic variables, MMSE scores, and medial temporal atrophy (MTA) measurements successfully classified 14 of 16 cognitively intact participants (87.5% sensitivity) and 72 of 82 ADD patients (87.8% specificity), yielding an overall diagnostic accuracy of 87.8%. Augmentation of this baseline model with plasma pTau181 significantly enhanced the overall classification accuracy to 90.8% (*p* < 0.05). Further refinement through the sequential addition of plasma pTau217 and the pTau217/181 ratio variables resulted in a small improvement in diagnostic accuracy to 95.1% (*p* < 0.01). The comprehensive model integrating demographic factors, cognitive assessment, neuroimaging metrics, and all plasma phosphorylated tau biomarkers demonstrated superior diagnostic performance, improving the correct classification rate of ADD patients from 87.8 to 95.4% (*p* < 0.001). Detailed statistical parameters of the stepwise linear discriminant analysis, including canonical correlations, Wilks’ lambda values, and classification matrices, are comprehensively presented in Table 4.

**TABLE 4:** Stepwise linear discriminant analysis to classify ADD/ADDvas patients and cognitively intact controls with and without blood based pTau biomarkers.

(a) Diagnostic classification matrix based on age, gender, MMSE total score, MTA score		
Classification	Predicted by LDA	
	Cognitively intact controls	AD/ADvas
Cognitively intact controls (*n* = 16)	**14 (87.5%)**	2 (12.5 %)
ADD/ADDvas (*n* = 82)	10 (12.2%)	**72 (87.8%)**
87.8% of cross-validated grouped cases were correctly classified		
**(b) Diagnostic classification matrix based on age, gender, MMSE total score, MTA score, AND pTau181**		
	**Predicted by LDA**	
	**Cognitively intact controls**	**AD/ADvas**
Cognitively intact controls (*n* = 16)	**15 (93.3%)**	1 (6.7 %)
ADD/ADDvas (*n* = 82)	8 (9.8%)	**74 (90.2%)**
90.8% of cross-validated grouped cases were correctly classified		
**(c) Diagnostic classification matrix based on age, gender, MMSE total score, MTA score, AND pTau217**		
	**Predicted by LDA**	
	**Cognitively intact controls**	**AD/ADvas**
Cognitively intact controls (*n* = 16)	**16 (100%)**	0 (0 %)
ADD/ADDvas (*n* = 65)	4 (6.2%)	**61 (93.8%)**
95.1% of cross-validated grouped cases were correctly classified		
**d) Diagnostic classification matrix based on age, gender, MMSE total score, MTA score, AND pTau181, pTau217, pTau 217/181 ratio**		
	**Predicted by LDA**	
	**Cognitively intact controls**	**AD/ADvas**
Cognitively intact controls (*n* = 16)	**16 (100%)**	0 (0 %)
ADD/ADDvas (*n* = 65)	3 (4.6%)	**62 (95.4%)**
95.1% of cross-validated grouped cases were correctly classified		

ADD, Alzheimer’s disease dementia; ADDvas, Alzheimer’s disease dementia with vascular pathology; MTA, medial temporal atrophy. Bold values indicate sensitivity and specificity.

### 3.7 Differential expression of plasma pTau based on biological biomarkers of AD and non-AD etiology

Comparison of plasma pTau’s and their ratio showed significantly higher concentrations of pTau217 and the pTau 217/181 ratio in Aß-PET positive compared to Aß-PET negative dementia patients (Table 5a). Similar results were obtained when comparing pTau plasma concentrations in ADD/ADDvas dementia and non-AD dementia patients (Table 5b). Comparison of plasma pTau’s and their ratio between patients with CSF-confirmed ADD and those without AD pathology in the CSF revealed significantly higher plasma pTau concentrations in the former group (Table 5c). In turn, patients with positive biomarkers for AD in the CSF and those with positive biomarkers in Aß-PET showed no significant differences in this regard (Table 5d). Group comparison of patients with ADD/ADDvas dementia showed significant higher values of pTau217 and the ratio compared to patients with non-AD dementia and comparable values for pTau181 (Table 5b). Due to the small number of participants in certain biomarker-defined subgroups, particularly the Amyloid negative group (*n* = 5) and the CSF normal group (*n* = 10), the statistical power is reduced, which limits the reliability and generalizability of the observed effects.

**TABLE 5a:** Comparison of pTau plasma concentrations in amyloid-positive (+) ADD patients and amyloid-negative (-) dementia patients of other etiology.

Variable	Diagnostic group of dementia patients		Test statistic ^a^		*p*-value
	Amyloid + *N* = 26	Amyloid - *N* = 5	*Z =*	*U =*	
pTau181 pg/mL	2.36 ± 0.95	2.22 ± 0.93	–0.295	59.5	0.775
pTau217 pg/mL	0.60 ± 0.38	0.23 ± 0.07	–2.392	5.0	0.011
pTau217/181 ratio	0.24 ± 0.08	0.13 ± 0.05	–2.546	3.0	< 0.005

^a^Due to deviations from a normal distribution the Mann-Whitney-U-Test was used.

**TABLE 5b:** Comparison of pTau plasma measures in ADD/ADDvas dementia and non-AD dementia patients.

Variable	Diagnostic group of dementia patients		Test statistic^a^		*p*-value
	ADD/ADDvas *N* = 94	Non AD *N* = 38	*Z* =	*U* =	
pTau181 pg/mL	2.44 ± 1.15	2.29 ± 1.53	–1.309	1525.5	0.190
pTau217 pg/mL	0.62 ± 0.41	0.29 ± 0.19	–4.075	419.0	< 0.001
pTau217/181 ratio	0.24 ± 0.08	0.15 ± 0.06	–4.953	310.0	< 0.001

^a^Due to deviations from a normal distribution the Mann-Whitney-U-Test was used. ADD, Alzheimer’s disease dementia.

**TABLE 5c:** Comparison of pTau plasma concentrations in patients with CSF-confirmed ADD and those without AD pathology in the CSF.

Variable	Diagnostic group of dementia patients		Test statistic^a^		*p*-value
	CSF: ADD + *N* = 21	CSF: normal *N* = 10	*Z* =	*U* =	
pTau181 pg/mL	2.92 ± 1.67	1.99 ± 2.01	–3.740	15.0	< 0.001
pTau217 pg/mL	0.80 ± 0.57	0.19 ± 0.17	–3.848	2.0	< 0.001
pTau217/181 ratio	0.25 ± 0.09	0.06 ± 0.02	–2.971	17.0	0.002
**CSF**					
Beta-Amyloid-42 pg/mL	377 ± 140	864 ± 344	–3.652	17.0	< 0.001
Total Tau pg/mL	741 ± 363	262 ± 84	–4.180	5.0	< 0.001
pTau181 pg/mL	112 ± 58	33 ± 10	–4.355	1.0	< 0.001
Ratio (Tau/Aβ42)	2.07 ± 1.32	0.32 ± 0.06	–4.359	0.0	< 0.001
Ratio (Aβ42/Aβ40)	0.04 ± 0.01	0.09 ± 0.01	–4.270	0.0	< 0.001

^a^Due to deviations from a normal distribution the Mann-Whitney-U-Test was used. ADD, Alzheimer’s disease dementia; CSF, cerebrospinal fluid.

**TABLE 5d:** Comparison of pTau plasma concentrations in dementia patients with CSF- and amyloid PET-confirmed ADD.

Variable	Diagnostic group of dementia patients		Test statistic^a^		*p*-value
	Amyloid + *N* = 26	CSF: ADD + *N* = 21	*Z* =	*U* =	
pTau181 pg/mL	2.36 ± 0.95	2.92 ± 1.67	–0.648	223.5	0.517
pTau217 pg/mL	0.60 ± 0.38	0.80 ± 0.57	–0.921	154.5	0.362
pTau217/181 ratio	0.24 ± 0.08	0.25 ± 0.09	–0.566	167.0	0.585

^a^Due to deviations from a normal distribution the Mann-Whitney-U-Test was used, ADD, Alzheimer’s dementia.

### 3.8 Plasma pTau levels and stability

**FIGURE 2:**
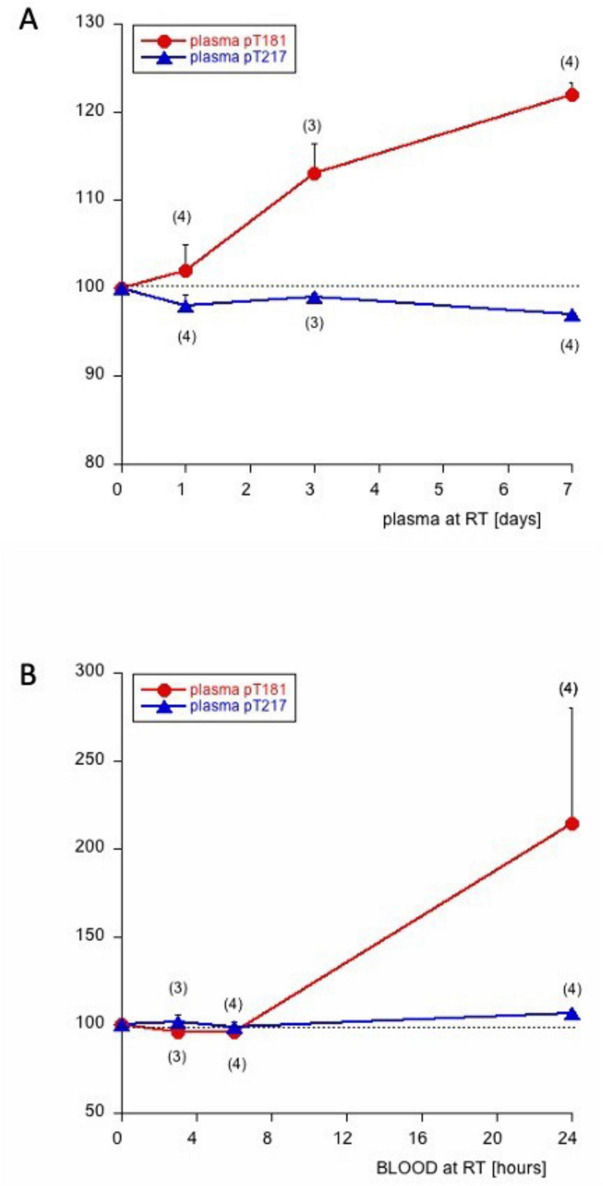
Stability of phospho-Tau (pTau) 181 and 217 in plasma **(A)** or whole blood **(B)**. Plasma was taken from 3 to 4 patients and stored for 0–1–3–7 days at room temperature (RT) and then analyzed **(A)** for pTau181 (red line, circles) or pTau 217 (blue line, triangles). Blood was collected from 3 to 4 patients and stored 0–3–6–24 h at room temperature, then centrifuged and analyzed **(B)**. Values in parentheses give the number of analyzed patients.

The Lumipulse assay is a very rapid and highly sensitive assay to quantify pTau levels in plasma. To validate analytical methodology and sample handling protocols, we conducted stability assessments. Plasma pTau217 demonstrated remarkable stability in plasma samples maintained at ambient temperature for up to 7 days (Figure 1A). In contrast, plasma pTau181 exhibited stability for only 24 h at room temperature, after which concentrations progressively increased, reaching approximately 120% of baseline values by day 7 (Figure 2A). pTau181 concentrations began to increase after just 6 h under identical conditions (Figure 2B). These differential stability profiles influenced our specimen handling protocol: all samples were analyzed on the day of collection when feasible; otherwise, plasma was stored at -20°C overnight before measurement to preserve biomarker integrity and ensure analytical consistency.

In control subjects (*n* = 43), mean plasma pTau181 concentration was 1.61 ± 0.73 pg/mL, while plasma pTau217 concentration was 0.19 ± 0.16 pg/mL (Table 2). The calculated ratio of plasma pTau217 to pTau181 was 0.14 ± 0.02 (Table 2).

## 4 Discussion

This study evaluated the clinical applicability of pTau181, pTau217, and their ratio measured by the Lumipulse immunoassay in a real-world memory clinic cohort with dementia of different etiologies and diagnostic certainty. The aim of this study was to test the accuracy of the two plasma tau markers and the newly introduced ratio for diagnosing or excluding ADD in routine clinical practice. We also wanted to provide reliable cut points for plasma tau markers to discriminate between people with clinically diagnosed dementia but no available biomarker, those with probable ADD, and cognitively healthy individuals. Our findings provide evidence that quantitative plasma tau measurements and the introduced pTau217/181 ratio demonstrate considerable sensitivity and specificity to serve as viable diagnostic biomarkers for Alzheimer’s disease, even in clinical contexts where comprehensive cerebrospinal fluid analysis or amyloid/tau positron emission tomography imaging is unavailable. However, the limited stability of pTau 181, and the resulting need for accurate processing, may give pTau 217 a significant advantage in routine clinical use.

### 4.1 Plasma pTau in cognitively intact and clinically diagnosed dementia of different etiologies

Our data show that plasma pTau181, pTau217, and their ratio can significantly discriminate between cognitively intact individuals and patients with a clinically evident dementia syndrome. Overall, the p-tau217/181 ratio showed AUC values comparable to p-tau217 alone, with only slightly higher specificity across both comparisons. Diagnostic accuracy was higher in the group of patients with probable ADD than in those without clinical evidence for AD. Although the newly introduced ratio demonstrated the greatest diagnostic utility, pTau217 alone performed reasonably well in distinguishing cognitively intact individuals, particularly from ADD patients. These findings were robust to validation using k-fold cross-validation and bootstrapping, ensuring stability and generalizability. This result suggests that pTau217 may possess superior specificity for AD-related pathophysiological processes compared to pTau181. Our results support those presented in Khalafi et al.’s review of 30 studies assessing the diagnostic accuracy of pTau 217 in distinguishing patients with AD from cognitively intact individuals (Khalafi et al., 2025). In contrast to our data, all included study populations had a biomarker assessment using amyloid PET or CSF, and were therefore not representative of a routine memory clinic population. Our findings suggest that pTau217 could be useful as a plasma-based biomarker for distinguishing between patients with ADD/ADDvas, patients with non-AD dementia, and cognitively intact individuals, in patients who have primarily been diagnosed clinically. We assume that plasma pTau217 offers a less invasive approach to the differential diagnosis of neurocognitive disorders in clinical settings.

### 4.2 Plasma pTau in patients with biomarker-diagnosed dementia

When all three pTau measures were compared between Aß PET-positive and Aß PET-negative dementia patients, pTau217 and the ratio were significantly higher in positive patients, but not pTau181. In contrast, all three plasma tau measures were higher in dementia patients with CSF-confirmed ADD than in dementia patients with no evidence of AD in the CSF. Comparison of CSF-confirmed and Aß PET-confirmed ADD showed no significant differences for all pTau measures. Our results are consistent with previous studies reporting superior diagnostic accuracy of pTau217 compared to pTau181 for identifying Aß-positive patients (Arranz et al., 2024). Nevertheless, our results must be interpreted with caution due to the small sample size. These limited group sizes reduce statistical power and restrict the reliability and generalizability of the observed effects. As such, the results from these subgroup comparisons should be interpreted as exploratory and descriptive. They primarily serve to generate hypotheses and to illustrate potential trends in plasma biomarker expression across reference modalities, rather than to support definitive conclusions. However, in accordance with earlier studies, pTau217 was able to distinguish between AD and non-AD dementia (Mielke et al., 2022, Khalafi et al., 2025, Palmqvist et al., 2025). In contrast to the analysis of a large real-world cohort by Cano et al. (Cano et al., 2024), we did not find a significant difference in terms of pTau181, but for the pTau217/181 ratio. Our data provide evidence that the combined assessment of both plasma pTau measures and their ratio may improve slightly their diagnostic validity in routine clinical practice. In support of this, we found that the combined use of pTau181, pTau217, and their ratio together with visual imaging assessment, age and the MMSE could correctly classify ADD with 95.4% accuracy. However, the small advantage of additionally analyzing pTau 181 may not outweigh the additional costs and disadvantages of plasma pTau 181, which is unstable and therefore requires immediate processing.

### 4.3 Plasma pTau marker cut-offs to diagnose ADD

For the clinical use of plasma pTau measures, it is essential to define pathological cut points that can differentiate between healthy aging and dementia and in particular AD. Our data show comparable cut points for pTau181, pTau217, and the ratio in a mixed sample of dementia patients and a more accurately diagnosed sample with AD. The diagnostic performance was slightly better than pTau 217 alone for the pTau217/181 ratio in ADD/ADDvas patients vs. cognitively intact subjects with an AUC of 0.91 and a cut point of 0.18. In agreement with a study in patients with MCI due to ADD (Lehmann et al., 2024), we found a cut point for pTau217 of 0.23 pg/mL to discriminate between cognitively intact subjects and ADD/ADDvas patients. Further, our results are in line with those of Cecchetti et al. (2024) who also found a cut point of 0.23 pg/mL for pTau217 when comparing biomarker positive ADD and non-AD patients. It has to be mentioned, however, that the sample included in that study was smaller and neither pTau 181 nor the pTau217/181 ratio was determined. In terms of pTau181, our cut point was 1.48 pg/mL, which is lower than that reported in a study by Lehmann et al. (2024). Nevertheless, the mean values in our biomarker-diagnosed ADD patients ranged from 2.36 to 2.92 pg/mL and were thus comparable to the cut point of 2.75 pg/mL reported by Lehmann and coworkers. Furthermore, the data are not fully comparable as Lehmann et al. used the Simoa technology.

Our data provide evidence that pTau217 is superior to pTau181 in distinguishing ADD from cognitively intact individuals or dementia of other etiologies. These results are in agreement with those of Martínez-Dubarbie et al. (2024) who reported a better diagnostic performance of pTau217 compared to pTau181 using the Lumipulse immunoassay with an AUC of 0.85 for detecting individuals with preclinical biomarker—positive AD. In contrast, compared to our findings, they reported much lower cut points of 0.095 pg/mL for pTau217 and 1.23 pg/mL for pTau181. Prior reports summarizing the results of studies on different cohorts reported distinct differences in cut points for pTau217, depending on the populations included in the studies (primary care, secondary care and specialized centers) ranging based on finite mixture modeling between 0.16 and 0.29 pg/mL (Liewand Alzheimer’s Disease Neuroimaging Initiative, 2025; Palmqvist et al., 2025). Due to the high rate of misclassification in patients with clinically diagnosed ADD, we assume that the cut points for pTau have to be set higher than for patients with biomarker-based diagnoses. Therefore, the reported cut point for pTau 217 (0.23 pg/mL) in our cohort of patients primarily diagnosed clinically might be suitable for routine clinical settings. Further, we hypothesize that plasma pTau accelerates from the preclinical to the clinical stages of AD. In support of this, Arranz et al. (2024) reported higher levels of pTau 217 and pTau181 measured by Lumipulse in AD compared with MCI and MCI compared with cognitively unimpaired Aß-positive individuals. However, we found no correlation between any of the plasma pTau measures and the MMSE as a measure of severity of cognitive impairment in ADD patients. Accordingly, we assume that plasma pTau, similar to Aß load or CSF markers, does not increase linearly when a critical threshold of cognitive decline is reached.

### 4.4 Plasma pTau for routine diagnostics

Taken together, our data provide evidence that plasma pTau217 alone and in combination with the pTau217/181 ratio are suitable for diagnosing ADD with high sensitivity and specificity in the absence of other amyloid or tau biomarkers measured by PET or CSF sampling. While p-tau217 alone demonstrated strong diagnostic performance, the p-tau217/181 ratio provided marginal improvements in specificity in both main group comparisons. Although sensitivity was slightly reduced in the ratio model, this trade-off might be acceptable in clinical settings where high specificity is critical. Given the close overlap of ROC curves, the added value of the ratio over p-tau217 alone appears limited but potentially relevant depending on the clinical use case. Future studies with independent cohorts should clarify whether this pattern persists.

Further, our data provide evidence that plasma pTau217 is stable for at least 3 days in plasma and 24 h in whole blood and can therefore easily be stored at room temperature and shipped to a laboratory. In turn, plasma pTau181 seems not to be as stable as pTau217 and the plasma sample must arrive in the lab within 24 h for valid analysis. It is not fully clear why the levels of plasma pTau181 markedly increase in plasma over time. However, one could speculate that plasma contains protein kinases, which specifically phosphorylate Tau-181 over time.

## 5 Limitations

Interpretation of our results is limited by the single-center design, the small number of participants with biomarker-diagnosed ADD and underpowered diagnostic categories of non-AD dementia. Therefore, the subgroup analysis of patients with a biomarker-diagnosed dementia (amyloid PET or CSF) can only be considered exploratory. Second, the DEM (132 patients with clinically diagnosed dementia) was heterogeneous, including 29% of patients with non-AD dementia and 71% of probable or possible ADD. Although this heterogeneity could affect statistical analyses, it is a very realistic representation of clinical reality. However, the cut points we have provided for the analyzed pTau measures cannot be generalized to other populations in the real world. In addition, we did not include participants with MCI or cognitively intact but biomarker-positive individuals. We did not conduct a blinded study comparing laboratory and clinical diagnoses, nor did we externally validate our presented cut-offs for pTau measures. In addition, we cannot exclude the possibility that various exogenous factors may have influenced plasma pTau levels, such as medication, smoking, circadian rhythm, hormone levels, or comorbidities. However, confounding factors such as acute infections or severely impaired renal function were excluded by routine blood sampling at the time of pTau assessment. Finally, we only studied white Caucasians and did not include other ethnicities.

## 6 Conclusion

This study was designed to evaluate the performance of plasma pTau217, pTau181, and the newly introduced pTau217/181 ratio in differentiating AD dementia and non-AD dementia as measured by the Lumipulse immunoassay in a real-world population. There is a rapidly growing body of evidence that both plasma pTau markers correlate well with Aß PET or CSF findings in preclinical or early clinical AD patients. However, in the real-world setting of memory clinics, the availability of the latter is limited and patients are mostly in the dementia stage at initial assessment. Our results showed that the combined use of plasma pTau217 and pTau181 and calculating the pTau 217/181 ratio, can discriminate between ADD and non-AD dementia in clinically diagnosed patients with a diagnostic accuracy of 95%. However, plasma pTau217 outperforms pTau181 and the ratio due to its greater stability and marginally higher sensitivity compared to the 217/181 ratio. In summary, based on our results, we conclude that using pTau217 as a simple and cost-effective method for assessing dementia can be useful in routine clinical practice. Plasma pTau217 levels may help define indications for further biomarker investigations using amyloid PET or CSF, although our data do not provide sufficient evidence to suggest that they can replace these methods.

## Data Availability

The original contributions presented in the study are included in the article/Supplementary material, further inquiries can be directed to the corresponding author.

## Footnotes

1
www.biofinder.se

2
https://www.fujirebio.com/en/products-solutions/lumipulse-g600ii

3
https://www.R-project.org/

4
https://CRAN.R-project.org/package=caret
