# Supplementary material for: Plasma phospho-tau 217 outperforms plasma phospho-tau 181 analyzed with Lumipulse in detecting Alzheimer’s dementia in a real-world memory clinic population
Source: Front Aging Neurosci. 2026 Feb 13;18:1714247. doi: 10.3389/fnagi.2026.1714247 (PMC12946082; doi:10.3389/fnagi.2026.1714247)
Supplement: Supplementary file 1 [file Data_Sheet_1.docx]

Supplement 1. Results of sensitivity analysis by summarizing the sensitivity and 1-specificity (false positive rate) for each threshold value between cognitively intact controls vs. ADD / ADDvas

| Cognitively intact controls vs. ADD / ADDvas | | | | | | | | | | |
| --- | --- | --- | --- | --- | --- | --- | --- | --- | --- | --- |
|  |  |  |  |  |  |  |  |  |  |  |
| **pTau181**  pg/ml | **Sensitivity** | **Specificity** |  | **pTau217**  pg/ml | **Sensitivity** | **Specificity** |  | **pTau 217/181 ratio** | **Sensitivity** | **Specificity** |
| INF | 100,00 | 0,00 |  | INF | 100,00 | 0,00 |  | INF | 100,00 | 0,00 |
| 0,565 | 100,00 | 2,38 |  | 0,061 | 100,00 | 3,85 |  | 0,051 | 100,00 | 3,85 |
| 0,710 | 100,00 | 4,76 |  | 0,073 | 100,00 | 7,69 |  | 0,061 | 98,65 | 3,85 |
| 0,775 | 98,94 | 4,76 |  | 0,081 | 100,00 | 11,54 |  | 0,067 | 98,65 | 7,69 |
| 0,820 | 98,94 | 7,14 |  | 0,085 | 100,00 | 15,38 |  | 0,069 | 98,65 | 11,54 |
| 0,850 | 98,94 | 9,52 |  | 0,089 | 100,00 | 19,23 |  | 0,070 | 98,65 | 15,38 |
| 0,880 | 98,94 | 11,90 |  | 0,091 | 100,00 | 23,08 |  | 0,072 | 98,65 | 19,23 |
| 0,915 | 97,87 | 11,90 |  | 0,093 | 100,00 | 26,92 |  | 0,075 | 98,65 | 23,08 |
| 0,945 | 97,87 | 14,29 |  | 0,099 | 98,65 | 26,92 |  | 0,078 | 98,65 | 26,92 |
| 0,965 | 97,87 | 16,67 |  | 0,104 | 98,65 | 30,77 |  | 0,079 | 98,65 | 30,77 |
| 1,005 | 96,81 | 16,67 |  | 0,106 | 98,65 | 34,62 |  | 0,080 | 98,65 | 34,62 |
| 1,035 | 96,81 | 19,05 |  | 0,110 | 98,65 | 38,46 |  | 0,084 | 98,65 | 38,46 |
| 1,055 | 95,74 | 19,05 |  | 0,122 | 97,30 | 38,46 |  | 0,091 | 98,65 | 42,31 |
| 1,075 | 95,74 | 23,81 |  | 0,131 | 97,30 | 42,31 |  | 0,095 | 98,65 | 46,15 |
| 1,110 | 95,74 | 26,19 |  | 0,133 | 95,95 | 42,31 |  | 0,104 | 98,65 | 50,00 |
| 1,155 | 94,68 | 26,19 |  | 0,136 | 94,59 | 42,31 |  | 0,113 | 97,30 | 50,00 |
| 1,185 | 94,68 | 30,95 |  | 0,142 | 94,59 | 46,15 |  | 0,114 | 97,30 | 53,85 |
| 1,205 | 93,62 | 30,95 |  | 0,148 | 94,59 | 50,00 |  | 0,115 | 97,30 | 57,69 |
| 1,215 | 93,62 | 35,71 |  | 0,150 | 94,59 | 53,85 |  | 0,116 | 95,95 | 57,69 |
| 1,230 | 93,62 | 38,10 |  | 0,166 | 93,24 | 53,85 |  | 0,117 | 94,59 | 57,69 |
| 1,250 | 93,62 | 47,62 |  | 0,185 | 93,24 | 61,54 |  | 0,119 | 94,59 | 61,54 |
| 1,270 | 93,62 | 50,00 |  | 0,191 | 91,89 | 61,54 |  | 0,121 | 93,24 | 61,54 |
| 1,285 | 93,62 | 52,38 |  | 0,194 | 90,54 | 61,54 |  | 0,126 | 93,24 | 65,38 |
| 1,340 | 92,55 | 52,38 |  | 0,197 | 90,54 | 65,38 |  | 0,134 | 93,24 | 69,23 |
| 1,395 | 92,55 | 54,76 |  | 0,198 | 89,19 | 65,38 |  | 0,141 | 93,24 | 73,08 |
| 1,415 | 90,43 | 54,76 |  | 0,204 | 89,19 | 69,23 |  | 0,144 | 91,89 | 73,08 |
| 1,435 | 89,36 | 57,14 |  | 0,215 | 87,84 | 73,08 |  | 0,145 | 90,54 | 73,08 |
| 1,455 | 87,23 | 57,14 |  | 0,222 | 86,49 | 73,08 |  | 0,146 | 90,54 | 76,92 |
| 1,475 | 87,23 | 64,29 |  | 0,226 | 86,49 | 76,92 |  | 0,150 | 89,19 | 76,92 |
| 1,485 | 86,17 | 64,29 |  | 0,230 | 86,49 | 84,62 |  | 0,154 | 87,84 | 76,92 |
| 1,495 | 85,11 | 64,29 |  | 0,260 | 85,14 | 84,62 |  | 0,157 | 87,84 | 80,77 |
| 1,505 | 84,04 | 64,29 |  | 0,290 | 83,78 | 84,62 |  | 0,161 | 86,49 | 80,77 |
| 1,515 | 82,98 | 64,29 |  | 0,296 | 82,43 | 84,62 |  | 0,162 | 86,49 | 84,62 |
| 1,525 | 80,85 | 64,29 |  | 0,305 | 82,43 | 88,46 |  | 0,164 | 85,14 | 84,62 |
| 1,555 | 79,79 | 64,29 |  | 0,315 | 81,08 | 88,46 |  | 0,168 | 83,78 | 84,62 |
| 1,590 | 78,72 | 64,29 |  | 0,321 | 79,73 | 88,46 |  | 0,171 | 83,78 | 88,46 |
| 1,605 | 77,66 | 64,29 |  | 0,326 | 78,38 | 88,46 |  | 0,176 | 82,43 | 88,46 |
| 1,615 | 76,60 | 64,29 |  | 0,331 | 77,03 | 88,46 |  | 0,178 | 81,08 | 88,46 |
| 1,625 | 74,47 | 64,29 |  | 0,340 | 77,03 | 92,31 |  | 0,179 | 81,08 | 92,31 |
| 1,635 | 72,34 | 64,29 |  | 0,349 | 75,68 | 92,31 |  | 0,182 | 79,73 | 92,31 |
| 1,645 | 71,28 | 64,29 |  | 0,360 | 74,32 | 92,31 |  | 0,183 | 78,38 | 92,31 |
| 1,655 | 70,21 | 64,29 |  | 0,372 | 72,97 | 92,31 |  | 0,184 | 77,03 | 92,31 |
| 1,680 | 68,09 | 64,29 |  | 0,377 | 71,62 | 92,31 |  | 0,184 | 77,03 | 96,15 |
| 1,720 | 67,02 | 64,29 |  | 0,387 | 70,27 | 92,31 |  | 0,185 | 75,68 | 96,15 |
| 1,750 | 64,89 | 64,29 |  | 0,395 | 68,92 | 92,31 |  | 0,186 | 74,32 | 96,15 |
| 1,775 | 63,83 | 64,29 |  | 0,408 | 67,57 | 92,31 |  | 0,187 | 72,97 | 96,15 |
| 1,810 | 62,77 | 64,29 |  | 0,420 | 66,22 | 92,31 |  | 0,188 | 71,62 | 96,15 |
| 1,860 | 61,70 | 64,29 |  | 0,425 | 64,86 | 92,31 |  | 0,191 | 70,27 | 96,15 |
| 1,905 | 61,70 | 66,67 |  | 0,437 | 63,51 | 92,31 |  | 0,193 | 68,92 | 96,15 |
| 1,930 | 60,64 | 66,67 |  | 0,447 | 62,16 | 92,31 |  | 0,196 | 67,57 | 96,15 |
| 1,945 | 59,57 | 66,67 |  | 0,451 | 59,46 | 92,31 |  | 0,203 | 66,22 | 96,15 |
| 1,960 | 58,51 | 66,67 |  | 0,454 | 56,76 | 92,31 |  | 0,206 | 64,86 | 96,15 |
| 1,980 | 58,51 | 69,05 |  | 0,457 | 55,41 | 92,31 |  | 0,209 | 63,51 | 96,15 |
| 2,000 | 56,38 | 69,05 |  | 0,464 | 55,41 | 96,15 |  | 0,213 | 62,16 | 96,15 |
| 2,025 | 56,38 | 73,81 |  | 0,471 | 52,70 | 96,15 |  | 0,215 | 60,81 | 96,15 |
| 2,050 | 55,32 | 73,81 |  | 0,482 | 51,35 | 96,15 |  | 0,219 | 59,46 | 96,15 |
| 2,065 | 53,19 | 73,81 |  | 0,494 | 50,00 | 96,15 |  | 0,224 | 58,11 | 96,15 |
| 2,075 | 52,13 | 73,81 |  | 0,498 | 48,65 | 96,15 |  | 0,225 | 56,76 | 96,15 |
| 2,105 | 51,06 | 73,81 |  | 0,515 | 45,95 | 96,15 |  | 0,226 | 55,41 | 96,15 |
| 2,140 | 48,94 | 73,81 |  | 0,532 | 44,59 | 96,15 |  | 0,227 | 54,05 | 96,15 |
| 2,160 | 47,87 | 73,81 |  | 0,535 | 43,24 | 96,15 |  | 0,227 | 52,70 | 96,15 |
| 2,175 | 47,87 | 76,19 |  | 0,539 | 41,89 | 96,15 |  | 0,228 | 51,35 | 96,15 |
| 2,190 | 46,81 | 76,19 |  | 0,566 | 40,54 | 96,15 |  | 0,228 | 50,00 | 96,15 |
| 2,205 | 44,68 | 78,57 |  | 0,617 | 39,19 | 96,15 |  | 0,230 | 48,65 | 96,15 |
| 2,220 | 43,62 | 78,57 |  | 0,652 | 37,84 | 96,15 |  | 0,231 | 47,30 | 96,15 |
| 2,235 | 42,55 | 78,57 |  | 0,664 | 33,78 | 96,15 |  | 0,233 | 45,95 | 96,15 |
| 2,250 | 42,55 | 80,95 |  | 0,669 | 32,43 | 96,15 |  | 0,237 | 44,59 | 96,15 |
| 2,270 | 41,49 | 80,95 |  | 0,684 | 31,08 | 96,15 |  | 0,240 | 43,24 | 96,15 |
| 2,320 | 41,49 | 83,33 |  | 0,709 | 29,73 | 96,15 |  | 0,242 | 41,89 | 96,15 |
| 2,365 | 40,43 | 83,33 |  | 0,744 | 27,03 | 96,15 |  | 0,243 | 40,54 | 96,15 |
| 2,380 | 39,36 | 83,33 |  | 0,789 | 25,68 | 96,15 |  | 0,244 | 39,19 | 96,15 |
| 2,400 | 38,30 | 83,33 |  | 0,830 | 24,32 | 96,15 |  | 0,245 | 37,84 | 96,15 |
| 2,425 | 37,23 | 83,33 |  | 0,880 | 24,32 | 100,00 |  | 0,246 | 36,49 | 96,15 |
| 2,490 | 37,23 | 88,10 |  | 0,913 | 22,97 | 100,00 |  | 0,255 | 35,14 | 96,15 |
| 2,545 | 36,17 | 88,10 |  | 0,918 | 21,62 | 100,00 |  | 0,262 | 33,78 | 96,15 |
| 2,555 | 35,11 | 88,10 |  | 0,925 | 20,27 | 100,00 |  | 0,263 | 32,43 | 96,15 |
| 2,590 | 34,04 | 88,10 |  | 0,931 | 18,92 | 100,00 |  | 0,266 | 31,08 | 96,15 |
| 2,660 | 32,98 | 88,10 |  | 0,933 | 17,57 | 100,00 |  | 0,270 | 29,73 | 96,15 |
| 2,745 | 30,85 | 88,10 |  | 0,962 | 16,22 | 100,00 |  | 0,273 | 28,38 | 96,15 |
| 2,795 | 29,79 | 88,10 |  | 0,995 | 14,86 | 100,00 |  | 0,279 | 27,03 | 96,15 |
| 2,820 | 29,79 | 90,48 |  | 1,055 | 13,51 | 100,00 |  | 0,284 | 25,68 | 96,15 |
| 2,875 | 29,79 | 95,24 |  | 1,120 | 12,16 | 100,00 |  | 0,290 | 24,32 | 96,15 |
| 2,915 | 28,72 | 95,24 |  | 1,140 | 10,81 | 100,00 |  | 0,296 | 22,97 | 96,15 |
| 2,925 | 28,72 | 97,62 |  | 1,190 | 9,46 | 100,00 |  | 0,300 | 21,62 | 96,15 |
| 2,955 | 27,66 | 97,62 |  | 1,237 | 6,76 | 100,00 |  | 0,305 | 20,27 | 96,15 |
| 3,005 | 26,60 | 97,62 |  | 1,312 | 5,41 | 100,00 |  | 0,307 | 18,92 | 96,15 |
| 3,055 | 25,53 | 97,62 |  | 1,485 | 4,05 | 100,00 |  | 0,308 | 17,57 | 96,15 |
| 3,095 | 24,47 | 97,62 |  | 1,710 | 2,70 | 100,00 |  | 0,326 | 16,22 | 96,15 |
| 3,115 | 23,40 | 97,62 |  | 2,080 | 1,35 | 100,00 |  | 0,346 | 14,86 | 96,15 |
| 3,145 | 21,28 | 97,62 |  | INF | 0,00 | 100,00 |  | 0,353 | 13,51 | 96,15 |
| 3,185 | 20,21 | 97,62 |  |  |  |  |  | 0,360 | 12,16 | 96,15 |
| 3,215 | 19,15 | 97,62 |  |  |  |  |  | 0,362 | 10,81 | 96,15 |
| 3,280 | 18,09 | 97,62 |  |  |  |  |  | 0,368 | 9,46 | 96,15 |
| 3,365 | 17,02 | 97,62 |  |  |  |  |  | 0,378 | 8,11 | 96,15 |
| 3,430 | 15,96 | 97,62 |  |  |  |  |  | 0,384 | 6,76 | 96,15 |
| 3,500 | 14,89 | 97,62 |  |  |  |  |  | 0,386 | 5,41 | 96,15 |
| 3,590 | 13,83 | 97,62 |  |  |  |  |  | 0,395 | 4,05 | 96,15 |
| 3,665 | 12,77 | 97,62 |  |  |  |  |  | 0,408 | 2,70 | 96,15 |
| 3,710 | 11,70 | 97,62 |  |  |  |  |  | 0,446 | 1,35 | 96,15 |
| 3,765 | 10,64 | 100,00 |  |  |  |  |  | 0,752 | 0,00 | 96,15 |
| 3,805 | 9,57 | 100,00 |  |  |  |  |  | INF | 0,00 | 100,00 |
| 3,875 | 8,51 | 100,00 |  |  |  |  |  |  |  |  |
| 4,195 | 7,45 | 100,00 |  |  |  |  |  |  |  |  |
| 4,490 | 6,38 | 100,00 |  |  |  |  |  |  |  |  |
| 4,680 | 5,32 | 100,00 |  |  |  |  |  |  |  |  |
| 5,025 | 4,26 | 100,00 |  |  |  |  |  |  |  |  |
| 5,620 | 3,19 | 100,00 |  |  |  |  |  |  |  |  |
| 6,035 | 2,13 | 100,00 |  |  |  |  |  |  |  |  |
| 6,365 | 1,06 | 100,00 |  |  |  |  |  |  |  |  |
| INF | 0,00 | 100,00 |  |  |  |  |  |  |  |  |

ADD = Alzheimer´s disease dementia, ADDvas = Alzheimer´s disease dementia with vascular pathology
