# Supplementary material for: Plasma phospho-tau 217 outperforms plasma phospho-tau 181 analyzed with Lumipulse in detecting Alzheimer’s dementia in a real-world memory clinic population
Source: Front Aging Neurosci. 2026 Feb 13;18:1714247. doi: 10.3389/fnagi.2026.1714247 (PMC12946082; doi:10.3389/fnagi.2026.1714247)
Supplement: Supplementary file 2 [file Data_Sheet_2.docx]

Supplement 2: Results of sensitivity analysis by summarizing the sensitivity and 1-specificity (false positive rate) for each threshold value between cognitively intact controls vs. clinically diagnosed dementia

| Cognitively intact controls vs. Clinically diagnosed dementia | | | | | | | | | | |
| --- | --- | --- | --- | --- | --- | --- | --- | --- | --- | --- |
|  |  |  |  |  |  |  |  |  |  |  |
| **pTau181**  pg/ml | **Sensitivity** | **Specificity** |  | **pTau217**  pg/ml | **Sensitivity** | **Specificity** |  | **pTau 217/181 ratio** | **Sensitivity** | **Specificity** |
| INF | 100,00 | 0,00 |  | INF | 100,00 | 0,00 |  | INF | 100,00 | 0,00 |
| 0,565 | 100,00 | 2,38 |  | 0,061 | 100,00 | 3,85 |  | 0,051 | 100,00 | 3,85 |
| 0,710 | 100,00 | 4,76 |  | 0,073 | 100,00 | 7,69 |  | 0,061 | 98,99 | 3,85 |
| 0,760 | 99,24 | 4,76 |  | 0,081 | 100,00 | 11,54 |  | 0,067 | 98,99 | 7,69 |
| 0,785 | 98,48 | 4,76 |  | 0,085 | 100,00 | 15,38 |  | 0,069 | 98,99 | 11,54 |
| 0,795 | 97,73 | 4,76 |  | 0,089 | 100,00 | 19,23 |  | 0,070 | 98,99 | 15,38 |
| 0,805 | 96,97 | 4,76 |  | 0,091 | 100,00 | 23,08 |  | 0,072 | 98,99 | 19,23 |
| 0,820 | 96,97 | 7,14 |  | 0,092 | 98,99 | 23,08 |  | 0,074 | 98,99 | 23,08 |
| 0,835 | 96,97 | 9,52 |  | 0,093 | 98,99 | 26,92 |  | 0,076 | 97,98 | 23,08 |
| 0,855 | 96,21 | 9,52 |  | 0,096 | 97,98 | 26,92 |  | 0,078 | 97,98 | 26,92 |
| 0,880 | 96,21 | 11,90 |  | 0,101 | 96,97 | 26,92 |  | 0,079 | 97,98 | 30,77 |
| 0,915 | 95,45 | 11,90 |  | 0,104 | 96,97 | 30,77 |  | 0,080 | 97,98 | 34,62 |
| 0,945 | 95,45 | 14,29 |  | 0,106 | 96,97 | 34,62 |  | 0,083 | 97,98 | 38,46 |
| 0,960 | 94,70 | 16,67 |  | 0,108 | 96,97 | 38,46 |  | 0,087 | 96,97 | 38,46 |
| 0,975 | 93,94 | 16,67 |  | 0,109 | 95,96 | 38,46 |  | 0,091 | 96,97 | 42,31 |
| 0,985 | 93,18 | 16,67 |  | 0,112 | 93,94 | 38,46 |  | 0,093 | 95,96 | 42,31 |
| 1,010 | 92,42 | 16,67 |  | 0,119 | 92,93 | 38,46 |  | 0,095 | 95,96 | 46,15 |
| 1,035 | 92,42 | 19,05 |  | 0,128 | 91,92 | 38,46 |  | 0,101 | 95,96 | 50,00 |
| 1,055 | 91,67 | 19,05 |  | 0,131 | 90,91 | 42,31 |  | 0,109 | 94,95 | 50,00 |
| 1,075 | 91,67 | 23,81 |  | 0,133 | 89,90 | 42,31 |  | 0,113 | 93,94 | 50,00 |
| 1,110 | 91,67 | 26,19 |  | 0,136 | 88,89 | 42,31 |  | 0,114 | 93,94 | 53,85 |
| 1,155 | 90,91 | 26,19 |  | 0,139 | 88,89 | 46,15 |  | 0,115 | 93,94 | 57,69 |
| 1,185 | 90,91 | 30,95 |  | 0,143 | 86,87 | 46,15 |  | 0,116 | 92,93 | 57,69 |
| 1,205 | 89,39 | 30,95 |  | 0,148 | 86,87 | 50,00 |  | 0,116 | 91,92 | 57,69 |
| 1,215 | 88,64 | 35,71 |  | 0,150 | 86,87 | 53,85 |  | 0,116 | 90,91 | 57,69 |
| 1,230 | 88,64 | 38,10 |  | 0,166 | 85,86 | 53,85 |  | 0,117 | 89,90 | 57,69 |
| 1,245 | 88,64 | 47,62 |  | 0,185 | 85,86 | 61,54 |  | 0,117 | 88,89 | 57,69 |
| 1,255 | 87,88 | 47,62 |  | 0,191 | 84,85 | 61,54 |  | 0,119 | 88,89 | 61,54 |
| 1,270 | 87,88 | 50,00 |  | 0,194 | 83,84 | 61,54 |  | 0,121 | 87,88 | 61,54 |
| 1,285 | 87,88 | 52,38 |  | 0,197 | 83,84 | 65,38 |  | 0,123 | 87,88 | 65,38 |
| 1,295 | 87,12 | 52,38 |  | 0,198 | 82,83 | 65,38 |  | 0,125 | 86,87 | 65,38 |
| 1,325 | 86,36 | 52,38 |  | 0,200 | 82,83 | 69,23 |  | 0,127 | 85,86 | 65,38 |
| 1,370 | 85,61 | 52,38 |  | 0,206 | 80,81 | 69,23 |  | 0,128 | 84,85 | 65,38 |
| 1,395 | 84,85 | 54,76 |  | 0,215 | 79,80 | 73,08 |  | 0,131 | 84,85 | 69,23 |
| 1,415 | 83,33 | 54,76 |  | 0,222 | 77,78 | 73,08 |  | 0,134 | 83,84 | 69,23 |
| 1,435 | 82,58 | 57,14 |  | 0,224 | 77,78 | 76,92 |  | 0,137 | 82,83 | 69,23 |
| 1,445 | 81,06 | 57,14 |  | 0,227 | 76,77 | 76,92 |  | 0,139 | 82,83 | 73,08 |
| 1,460 | 80,30 | 57,14 |  | 0,230 | 76,77 | 84,62 |  | 0,141 | 81,82 | 73,08 |
| 1,475 | 80,30 | 64,29 |  | 0,240 | 75,76 | 84,62 |  | 0,144 | 80,81 | 73,08 |
| 1,485 | 79,55 | 64,29 |  | 0,268 | 74,75 | 84,62 |  | 0,145 | 79,80 | 73,08 |
| 1,495 | 78,79 | 64,29 |  | 0,288 | 73,74 | 84,62 |  | 0,146 | 78,79 | 73,08 |
| 1,505 | 78,03 | 64,29 |  | 0,290 | 71,72 | 84,62 |  | 0,146 | 78,79 | 76,92 |
| 1,515 | 76,52 | 64,29 |  | 0,296 | 70,71 | 84,62 |  | 0,148 | 77,78 | 76,92 |
| 1,525 | 75,00 | 64,29 |  | 0,305 | 70,71 | 88,46 |  | 0,150 | 76,77 | 76,92 |
| 1,555 | 74,24 | 64,29 |  | 0,315 | 69,70 | 88,46 |  | 0,152 | 75,76 | 76,92 |
| 1,590 | 73,48 | 64,29 |  | 0,321 | 68,69 | 88,46 |  | 0,154 | 74,75 | 76,92 |
| 1,605 | 72,73 | 64,29 |  | 0,326 | 67,68 | 88,46 |  | 0,155 | 74,75 | 80,77 |
| 1,615 | 71,97 | 64,29 |  | 0,331 | 66,67 | 88,46 |  | 0,157 | 73,74 | 80,77 |
| 1,625 | 70,45 | 64,29 |  | 0,340 | 66,67 | 92,31 |  | 0,160 | 72,73 | 80,77 |
| 1,635 | 68,94 | 64,29 |  | 0,349 | 65,66 | 92,31 |  | 0,161 | 71,72 | 80,77 |
| 1,645 | 68,18 | 64,29 |  | 0,360 | 64,65 | 92,31 |  | 0,162 | 71,72 | 84,62 |
| 1,655 | 66,67 | 64,29 |  | 0,371 | 63,64 | 92,31 |  | 0,162 | 70,71 | 84,62 |
| 1,680 | 65,15 | 64,29 |  | 0,373 | 62,63 | 92,31 |  | 0,165 | 69,70 | 84,62 |
| 1,720 | 64,39 | 64,29 |  | 0,375 | 61,62 | 92,31 |  | 0,168 | 68,69 | 84,62 |
| 1,750 | 62,12 | 64,29 |  | 0,378 | 60,61 | 92,31 |  | 0,170 | 68,69 | 88,46 |
| 1,775 | 61,36 | 64,29 |  | 0,387 | 59,60 | 92,31 |  | 0,173 | 67,68 | 88,46 |
| 1,810 | 60,61 | 64,29 |  | 0,395 | 58,59 | 92,31 |  | 0,176 | 66,67 | 88,46 |
| 1,860 | 59,85 | 64,29 |  | 0,408 | 57,58 | 92,31 |  | 0,178 | 65,66 | 88,46 |
| 1,905 | 59,09 | 66,67 |  | 0,420 | 56,57 | 92,31 |  | 0,179 | 65,66 | 92,31 |
| 1,930 | 58,33 | 66,67 |  | 0,425 | 54,55 | 92,31 |  | 0,182 | 64,65 | 92,31 |
| 1,945 | 57,58 | 66,67 |  | 0,437 | 53,54 | 92,31 |  | 0,183 | 63,64 | 92,31 |
| 1,960 | 56,82 | 66,67 |  | 0,447 | 52,53 | 92,31 |  | 0,184 | 62,63 | 92,31 |
| 1,980 | 56,82 | 69,05 |  | 0,451 | 50,51 | 92,31 |  | 0,184 | 62,63 | 96,15 |
| 2,000 | 55,30 | 69,05 |  | 0,454 | 48,48 | 92,31 |  | 0,185 | 61,62 | 96,15 |
| 2,020 | 55,30 | 73,81 |  | 0,457 | 47,47 | 92,31 |  | 0,186 | 60,61 | 96,15 |
| 2,035 | 54,55 | 73,81 |  | 0,464 | 47,47 | 96,15 |  | 0,187 | 59,60 | 96,15 |
| 2,050 | 53,79 | 73,81 |  | 0,471 | 45,45 | 96,15 |  | 0,188 | 58,59 | 96,15 |
| 2,065 | 52,27 | 73,81 |  | 0,480 | 43,43 | 96,15 |  | 0,190 | 57,58 | 96,15 |
| 2,075 | 51,52 | 73,81 |  | 0,491 | 42,42 | 96,15 |  | 0,192 | 56,57 | 96,15 |
| 2,105 | 50,76 | 73,81 |  | 0,494 | 41,41 | 96,15 |  | 0,193 | 55,56 | 96,15 |
| 2,140 | 49,24 | 73,81 |  | 0,498 | 40,40 | 96,15 |  | 0,196 | 54,55 | 96,15 |
| 2,160 | 48,48 | 73,81 |  | 0,515 | 38,38 | 96,15 |  | 0,203 | 53,54 | 96,15 |
| 2,175 | 47,73 | 76,19 |  | 0,532 | 37,37 | 96,15 |  | 0,206 | 52,53 | 96,15 |
| 2,190 | 46,97 | 76,19 |  | 0,535 | 36,36 | 96,15 |  | 0,209 | 51,52 | 96,15 |
| 2,205 | 44,70 | 78,57 |  | 0,539 | 35,35 | 96,15 |  | 0,213 | 50,51 | 96,15 |
| 2,220 | 43,94 | 78,57 |  | 0,546 | 34,34 | 96,15 |  | 0,215 | 49,49 | 96,15 |
| 2,235 | 43,18 | 78,57 |  | 0,570 | 32,32 | 96,15 |  | 0,218 | 48,48 | 96,15 |
| 2,250 | 43,18 | 80,95 |  | 0,617 | 31,31 | 96,15 |  | 0,221 | 47,47 | 96,15 |
| 2,270 | 42,42 | 80,95 |  | 0,652 | 30,30 | 96,15 |  | 0,224 | 46,46 | 96,15 |
| 2,315 | 42,42 | 83,33 |  | 0,664 | 27,27 | 96,15 |  | 0,225 | 45,45 | 96,15 |
| 2,355 | 41,67 | 83,33 |  | 0,669 | 26,26 | 96,15 |  | 0,226 | 44,44 | 96,15 |
| 2,365 | 40,91 | 83,33 |  | 0,684 | 25,25 | 96,15 |  | 0,227 | 43,43 | 96,15 |
| 2,380 | 39,39 | 83,33 |  | 0,709 | 24,24 | 96,15 |  | 0,227 | 42,42 | 96,15 |
| 2,400 | 38,64 | 83,33 |  | 0,730 | 22,22 | 96,15 |  | 0,228 | 41,41 | 96,15 |
| 2,415 | 37,88 | 83,33 |  | 0,749 | 21,21 | 96,15 |  | 0,228 | 40,40 | 96,15 |
| 2,430 | 37,12 | 83,33 |  | 0,763 | 20,20 | 96,15 |  | 0,230 | 39,39 | 96,15 |
| 2,455 | 37,12 | 88,10 |  | 0,789 | 19,19 | 96,15 |  | 0,231 | 38,38 | 96,15 |
| 2,505 | 36,36 | 88,10 |  | 0,830 | 18,18 | 96,15 |  | 0,231 | 37,37 | 96,15 |
| 2,545 | 35,61 | 88,10 |  | 0,880 | 18,18 | 100,00 |  | 0,233 | 36,36 | 96,15 |
| 2,555 | 34,85 | 88,10 |  | 0,913 | 17,17 | 100,00 |  | 0,237 | 35,35 | 96,15 |
| 2,575 | 34,09 | 88,10 |  | 0,918 | 16,16 | 100,00 |  | 0,240 | 34,34 | 96,15 |
| 2,605 | 33,33 | 88,10 |  | 0,925 | 15,15 | 100,00 |  | 0,242 | 33,33 | 96,15 |
| 2,660 | 32,58 | 88,10 |  | 0,931 | 14,14 | 100,00 |  | 0,243 | 32,32 | 96,15 |
| 2,705 | 31,06 | 88,10 |  | 0,933 | 13,13 | 100,00 |  | 0,244 | 31,31 | 96,15 |
| 2,750 | 30,30 | 88,10 |  | 0,962 | 12,12 | 100,00 |  | 0,245 | 30,30 | 96,15 |
| 2,795 | 29,55 | 88,10 |  | 0,995 | 11,11 | 100,00 |  | 0,246 | 29,29 | 96,15 |
| 2,820 | 28,79 | 90,48 |  | 1,055 | 10,10 | 100,00 |  | 0,255 | 28,28 | 96,15 |
| 2,875 | 28,79 | 95,24 |  | 1,12 | 9,09 | 100,00 |  | 0,262 | 27,27 | 96,15 |
| 2,915 | 28,03 | 95,24 |  | 1,14 | 8,08 | 100,00 |  | 0,263 | 26,26 | 96,15 |
| 2,925 | 28,03 | 97,62 |  | 1,19 | 7,07 | 100,00 |  | 0,264 | 25,25 | 96,15 |
| 2,955 | 27,27 | 97,62 |  | 1,237 | 5,05 | 100,00 |  | 0,266 | 24,24 | 96,15 |
| 2,985 | 26,52 | 97,62 |  | 1,312 | 4,04 | 100,00 |  | 0,270 | 23,23 | 96,15 |
| 3,000 | 25,76 | 97,62 |  | 1,485 | 3,03 | 100,00 |  | 0,273 | 22,22 | 96,15 |
| 3,020 | 25,00 | 97,62 |  | 1,71 | 2,02 | 100,00 |  | 0,279 | 21,21 | 96,15 |
| 3,055 | 24,24 | 97,62 |  | 2,08 | 1,01 | 100,00 |  | 0,284 | 20,20 | 96,15 |
| 3,095 | 23,48 | 97,62 |  | INF | 0,00 | 100,00 |  | 0,290 | 19,19 | 96,15 |
| 3,115 | 22,73 | 97,62 |  |  |  |  |  | 0,296 | 18,18 | 96,15 |
| 3,125 | 20,45 | 97,62 |  |  |  |  |  | 0,300 | 17,17 | 96,15 |
| 3,150 | 19,70 | 97,62 |  |  |  |  |  | 0,305 | 16,16 | 96,15 |
| 3,185 | 18,94 | 97,62 |  |  |  |  |  | 0,307 | 15,15 | 96,15 |
| 3,215 | 18,18 | 97,62 |  |  |  |  |  | 0,308 | 14,14 | 96,15 |
| 3,28 | 17,42 | 97,62 |  |  |  |  |  | 0,321 | 13,13 | 96,15 |
| 3,355 | 16,67 | 97,62 |  |  |  |  |  | 0,338 | 12,12 | 96,15 |
| 3,39 | 15,91 | 97,62 |  |  |  |  |  | 0,346 | 11,11 | 96,15 |
| 3,42 | 15,15 | 97,62 |  |  |  |  |  | 0,353 | 10,10 | 96,15 |
| 3,45 | 14,39 | 97,62 |  |  |  |  |  | 0,360 | 9,09 | 96,15 |
| 3,5 | 12,88 | 97,62 |  |  |  |  |  | 0,362 | 8,08 | 96,15 |
| 3,555 | 12,12 | 97,62 |  |  |  |  |  | 0,368 | 7,07 | 96,15 |
| 3,605 | 11,36 | 97,62 |  |  |  |  |  | 0,378 | 6,06 | 96,15 |
| 3,665 | 10,61 | 97,62 |  |  |  |  |  | 0,384 | 5,05 | 96,15 |
| 3,71 | 9,85 | 97,62 |  |  |  |  |  | 0,386 | 4,04 | 96,15 |
| 3,765 | 9,09 | 100,00 |  |  |  |  |  | 0,395 | 3,03 | 96,15 |
| 3,805 | 8,33 | 100,00 |  |  |  |  |  | 0,408 | 2,02 | 96,15 |
| 3,875 | 7,58 | 100,00 |  |  |  |  |  | 0,446 | 1,01 | 96,15 |
| 4,195 | 6,82 | 100,00 |  |  |  |  |  | 0,752 | 0,00 | 96,15 |
| 4,49 | 6,06 | 100,00 |  |  |  |  |  | INF | 0,00 | 100,00 |
| 4,68 | 5,30 | 100,00 |  |  |  |  |  |  |  |  |
| 5,025 | 4,55 | 100,00 |  |  |  |  |  |  |  |  |
| 5,62 | 3,79 | 100,00 |  |  |  |  |  |  |  |  |
| 6,035 | 3,03 | 100,00 |  |  |  |  |  |  |  |  |
| 6,24 | 2,27 | 100,00 |  |  |  |  |  |  |  |  |
| 6,555 | 1,52 | 100,00 |  |  |  |  |  |  |  |  |
| 7,6 | 0,76 | 100,00 |  |  |  |  |  |  |  |  |
| INF | 0,00 | 100,00 |  |  |  |  |  |  |  |  |
|  |  |  |  |  |  |  |  |  |  |  |
