# Supplementary material for: Plasma phospho-tau 217 outperforms plasma phospho-tau 181 analyzed with Lumipulse in detecting Alzheimer’s dementia in a real-world memory clinic population
Source: Front Aging Neurosci. 2026 Feb 13;18:1714247. doi: 10.3389/fnagi.2026.1714247 (PMC12946082; doi:10.3389/fnagi.2026.1714247)
Supplement: Supplementary file 3 [file Data_Sheet_3.docx]

Supplement 3

Correlation of p-tau biomarkers with age, MMSE, and Apo ɛ genotype in patients with Alzheimer´s dementia (ADD/ADDvas)

| Spearman correlation | | | | | | | |
| --- | --- | --- | --- | --- | --- | --- | --- |
| Variable |  | 1 | 2 | 3 | 4 | 5 | 6 |
| 1. Age (years) | n | 94 |  |  |  |  |  |
| 2. MMSE total score | *Spearman r* | ,140 | -- |  |  |  |  |
|  | *p*-value | ,179 | . |  |  |  |  |
| 3. Apo ɛ genotype^a^ | *Spearman r* | -,208 | -,164 | -- |  |  |  |
|  | *p*-value | ,067 | ,152 | . |  |  |  |
| 4. pTau181 pg/mL | *Spearman r* | ,097 | -,199 | ,324 | -- |  |  |
|  | *p*-value | ,351 | ,055 | ,004 | . |  |  |
| 5. pTau217 pg/mL^b^ | *Spearman r* | -,018 | -,126 | ,237 | ,870 | -- |  |
|  | *p*-value | ,880 | ,287 | ,066 | <,001 | . |  |
| 6. pTau217/181 ratio^b^ | *Spearman r* | -,093 | -,146 | ,193 | ,356 | ,739 | -- |
|  | *p*-value | ,432 | ,216 | ,136 | ,002 | <,001 | . |

Abbreviations: ADD = Alzheimer’s dementia, ADDvas = Alzheimer´s dementia with vascular pathology, MMSE= Mini-Mental State Examination

^a^ number of Apo ɛ 4 allele (0, 1, or 2)

^b^ N= 74
