# Supplementary material for: Plasma phospho-tau 217 outperforms plasma phospho-tau 181 analyzed with Lumipulse in detecting Alzheimer’s dementia in a real-world memory clinic population
Source: Front Aging Neurosci. 2026 Feb 13;18:1714247. doi: 10.3389/fnagi.2026.1714247 (PMC12946082; doi:10.3389/fnagi.2026.1714247)
Supplement: Supplementary file 4 [file Table_1.docx]

**Specification of Logistic Regression Models**

The following logistic regression models were used to evaluate the association between plasma p-tau biomarkers and diagnostic group membership (i.e. CIC vs. ADD/ADDvas or CIC vs. DEM). The dependent variable Y was binary-coded, where:

$Y=1$: Alzheimer´s disease dementia without/with vascular pathology (ADD/ADDvas) or Dementia patients (DEM)

$Y=0$: Cognitively intact controls (CIC)

The logit link function was defined as:

$$\mathrm{logit}\left( P \right) = \log\left( \frac{P}{1-P} \right)$$

The intercept $\beta_{0}$; and the regression coefficient of the biomarker $\beta_{1}$

The models were specified as follows:

**Model 1: p-tau 181 (CIC vs ADD/ADDvas)**

$$\mathrm{logit}\left( P\left( Y=1 \right) \right)= \beta_{0}+ \beta_{1}\times pTau 181$$

**Model 2: p-tau 217 (CIC vs. ADD/ADDvas)**

$$\mathrm{logit}\left( P\left( Y=1 \right) \right)= \beta_{0}+ \beta_{1}\times pTau 217$$

**Model 3: p-tau 217/181 ratio (CIC vs. ADD/ADDvas)**

$$\mathrm{logit}\left( P\left( Y=1 \right) \right)= \beta_{0}+ \beta_{1}\times\left( \frac{pTau 217}{pTau 181} \right)$$

**Model 4: p-tau 181 (CIC vs. DEM)**

$$\mathrm{logit}\left( P\left( Y=1 \right) \right)= \beta_{0}+ \beta_{1}\times pTau 181$$

**Model 5: p-tau 217 (CIC vs. DEM)**

$$\mathrm{logit}\left( P\left( Y=1 \right) \right)= \beta_{0}+ \beta_{1}\times pTau 217$$

**Model 6: p-tau 217/181 ratio (CIC vs. DEM)**

$$\mathrm{logit}\left( P\left( Y=1 \right) \right)= \beta_{0}+ \beta_{1}\times\left( \frac{pTau 217}{pTau 181} \right)$$
